# Study of the gut microbiome in Egyptian patients with Parkinson’s Disease

**DOI:** 10.1186/s12866-023-02933-7

**Published:** 2023-07-22

**Authors:** Mohammad Mehanna, Suzan AbuRaya, Shwikar Mahmoud Ahmed, Ghada Ashmawy, Ahmed Ibrahim, Essameldin AbdelKhaliq

**Affiliations:** 1 Internal Medicine, Faculty of Medicine, University of Alexandria, Alexandria, Egypt; 2 Medical Microbiology and Immunology Department, Faculty of Medicine, University of Alexandria, Alexandria, Egypt; 3 Department of Neuropsychiatry, Faculty of Medicine, University of Alexandria, Alexandria, Egypt

**Keywords:** Parkinson’s disease, Gut microbiome, Brain-gut axis, Real-time PCR, 16S rRNA, Dysbiosis, Clinical phenotypes

## Abstract

**Background:**

Recently, an important relationship between Parkinson’s disease and the gut microbiota, through the brain-gut axis interactions, has been established. Previous studies have declared that alterations in the gut microbiota have a great impact on the pathogenesis and clinical picture of Parkinson’s disease (PD). The present study aimed to identify the gut microbiome that is likely related to Parkinson’s disease as well as their possible relation to clinical phenotypes.

**Methods:**

Thirty patients with Parkinson’s disease, who presented to the Parkinson’s disease Neurology Clinic of Alexandria University Hospital were enrolled in our study. A cross-matching control group of 35 healthy subjects of similar age and sex were included. Stool specimens were taken from each. Quantitative SYBR Green Real-Time PCR was done for the identification and quantitation of selected bacterial phyla, genera and/or species.

**Results:**

There was a significant increase in *Bacteroides* and a significant decrease of *Firmicutes* and *Firmicutes* / *Bacteroidetes* ratio and *Bifidobacteria* in PD patients. Although *Prevotella* was decreased among PD patients relative to the healthy control, the difference was not statistically significant. Comparing the PD clinical phenotypes with the control group, the Mixed phenotype had significantly higher *Bacteroides*, Tremors predominant had lower *Firmicutes* and *Firmicutes* / *Bacteroidetes* ratio, and both tremors and postural instability and gait disability (PIGD) phenotypes had lower *Bifidobacteria*. However, there was no statistically significant difference between these phenotypes. Furthermore, when comparing tremors and non-tremors predominant phenotypes; *Lactobacilli* showed a significant decrease in non-tremors predominant phenotypes.

**Conclusions:**

The current study showed evidence of changes in the gut microbiome of Parkinson’s disease patients compared to the healthy controls. These observations may highlight the importance of the identification of microbiome and specific bacterial changes that can be targeted for the treatment of Parkinson’s disease.

**Supplementary Information:**

The online version contains supplementary material available at 10.1186/s12866-023-02933-7.

## Introduction

Parkinson’s disease (PD) is a common and important neurodegenerative disease that affects many people all over the world [1, 2]. PD can be clinically defined by specific motor manifestations like rest tremor, rigidity, bradykinesia, and postural instability. However, non­motor manifestations of PD, like sleep disturbance, depression, gastrointestinal dysmotility, and constipation, represent an important category of the disease and may precede motor manifestations by years [3, 4].

Patients with Parkinson’s disease can be classified into Tremors-dominant subtype and postural instability and gait disability subtype (PIGD); based on the expression of tremors and PIGD, using relative subscores of the Unified Parkinson’s Disease Rating Scale (UPDRS). Groups that have mixed features from both are named Mixed subtype [3, 5].

The pathologic hallmark of PD is the gradual aggregation of misfolded alpha- synuclein (α-Syn) protein in the form of intracytoplasmic inclusions (Lewy bodies) in the nervous system [6]. Lewy bodies causes degeneration and loss of dopamine-producing neurons in the substantia nigra pars compacta of the midbrain which controls movement [3]. Current hypotheses suggest that the enteric nervous system (ENS) might be one of the first sites where Lewy body pathology appears in PD. Gastrointestinal symptoms, as well as Lewy body pathology in the ENS occur at the early stages of PD, suggesting that the gut is the origin of the pathological process that underlies PD [6, 7].

An important relationship between Parkinson’s disease and the gut microbiota, through the brain-gut axis interactions, has been established. Changes in the gut microbiota composition may cause alterations in the gut barrier function and intestinal permeability, affecting not only gut epithelial cells and the immune system, but also the ENS cells. The bidirectional brain-gut microbiota axis interactions modulate pro- and anti-inflammatory responses. It has been suggested that the gut microbiota changes associated with intestinal inflammation may contribute to the initiation of α-synuclein misfolding [8, 9].

The gut microbiota has recently emerged as a major topic of medical research. Previous studies have declared that alterations in the gut microbiota have a great impact on the pathogenesis and clinical picture of Parkinson’s Disease [10–12]. The first study on the alterations in the gut microbiota composition in PD and its association with the clinical phenotype of the disease showed a reduced abundance of the *Prevotella* in PD patients compared with healthy controls and among the PIGD phenotype compared to those with tremors-dominant PD patients [10].

*Prevotella* bacteria as commensals are involved in mucin synthesis in the gut mucosal layer and production of neuroactive short-chain fatty acids (SCFA) through fiber fermentation [13]. Thus the reduced abundance of *Prevotella* could result in decreased mucin synthesis and increased intestinal permeability leading to the greater local and systemic exposure to bacterial antigens and endotoxins, which in turn would initiate excessive α-syn expression or even promote its misfolding in ENS and its propagation to the central nervous system (CNS) via the vagus nerve in prion-like fashion [14–16].

Further studies on PD microbiome in different geographical areas are needed to highlight its possible role in disease pathophysiology. The present study aimed to identify the gut microbiome that is likely related to PD as well as their possible relation to clinical phenotypes and disease severity. This understanding may help in the development of new approaches for the treatment and prevention of PD by modulating the gut microbiome.

## Results

### Demographic and clinical data of cases

Demographic data revealed that out of the 30 PD patients, 22 patients (73.3%) were males and 8 patients (26.7%) were females, with a male to female ratio of 2.8:1. In the control group, 21 subjects (60%) were males and 14 subjects (40%) were females with a male to female ratio of 1.5:1. In the PD group, patients’ age ranged from 60 to 80 years with a mean age of 65.47 ± 6.10 years, while in the control group age ranged from 60 to 81 years with a mean age of 64.83 ± 5.63 years. The mean BMI (kg/m2) ± Standard Deviation (SD) of cases was 28.30 ± 3.97, while that of the controls was 30.29 ± 4.26.

All the 30 PD patients were on dopaminergic drugs. The mean disease duration for PD (defined by the time the first motor symptoms were experienced by the patient) was 7.50 ± 6.64 years (range: 1–24 years) with 19 cases having a disease duration of < 10 years and 11 cases with ≥ 10 years duration.

The clinical phenotypes were 21 (70%) tremors dominant (TD), 6 (20%) postural instability and gait difficulty (PIGD), and 3 (10%) mixed phenotypes (MX). The median UPDRS and NMS severity scores were 46.5 and 51.5 respectively. According to the modified Hoehn & Yahr scale; 25 (83.3%) of PD cases were mild and 5 (16.3%) were severe.

According to Wexner Score; 9 (30%), 3 (10%), 10 (33.3%), and 8 (26.7%) of PD cases have mild, moderate, severe, and very severe constipation respectively. For the control group 25 (71.4%), 7 (20%), 2 (5.7%), and 1 (2.9%) subjects have mild, moderate, severe, and very severe constipation respectively, with significantly more constipation severity in PD cases.

The different variables of Wexner Score in both cases and controls are shown in Table (S1). There was a statistically significant difference between the two groups as regards the total Wexner Constipation Score (p < 0.001). Also, all the variables were statistically significant more severe in PD cases than in control, except for the frequency of bowel movement and duration of constipation (p < 0.001).

### Gut microbiome analysis

Quantitation of specific bacteria DNA was not expressed as an absolute number but was expressed relative to the total bacteria DNA present in the stool sample.

#### Phylum level analysis

**Fig. 1:**
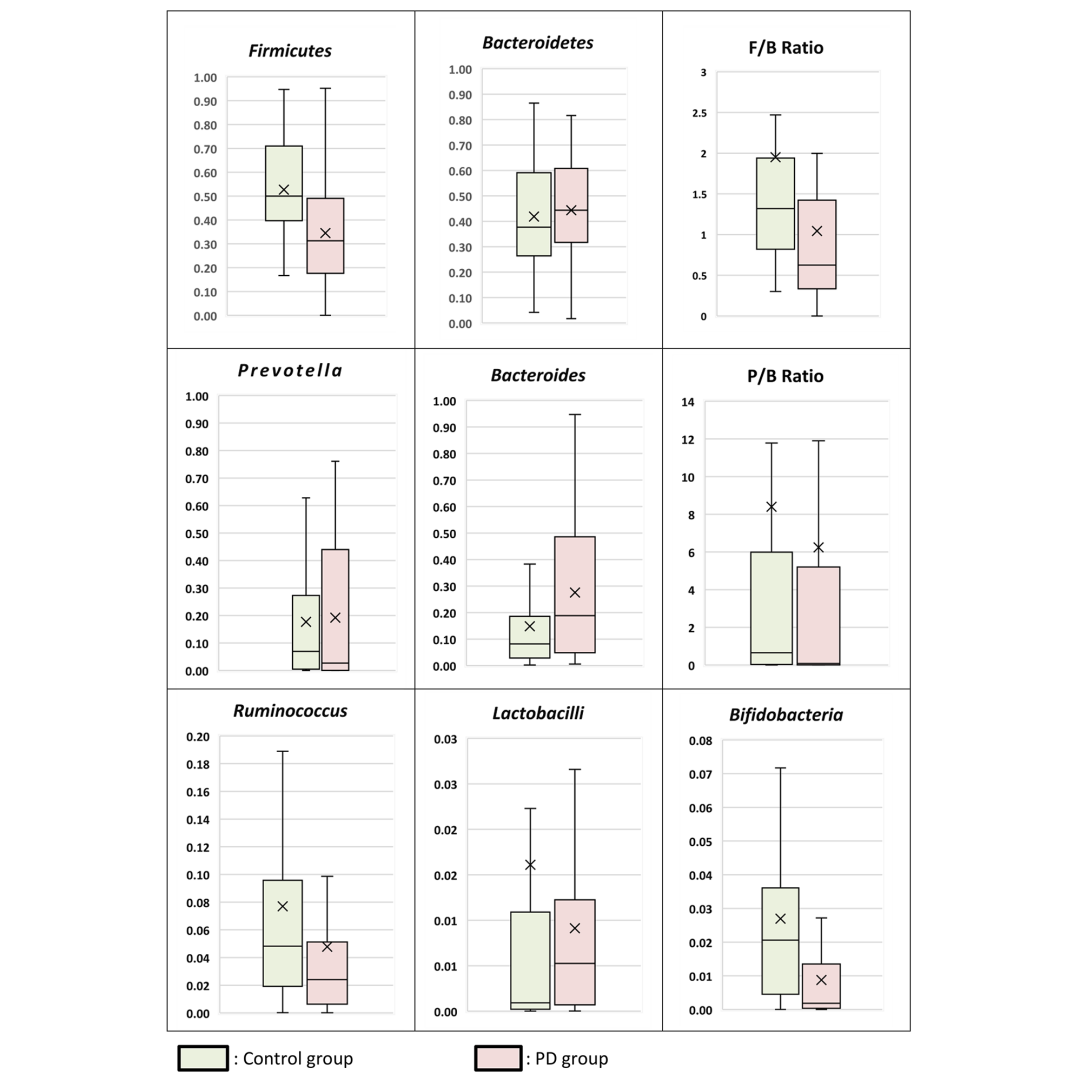
Gut Microbiome Profile of PD and Control Groups

Bacterial phylum analysis showed that patients with PD showed a statistically significant decrease in *Firmicutes* (p = 0.001) and although the *Bacteroidetes* was increased, the difference was not statistically significant in comparison to the control group. As regards the F/B ratio it was significantly lower in PD patients; median of 0.62 in PD cases versus 1.32 in control subjects (p = 0.003) (Table 1; Fig. 1).

#### Genus level analysis

Patients with PD showed a statistically significant increase in *Bacteroides* in comparison to the control group (P = 0.026). Meanwhile, there was no statistically significant difference between PD patients and control cases as regards *Prevotella* and *Ruminococcus.* Also, there was no statistically significant difference between PD and the control group as regards the *Prevotella/Bacteroides* Ratio (P/B) (Table 1; Fig. 1).

#### Species-level analysis

As regards the beneficial bacteria; patients with PD showed a statistically significant decrease in *Bifidobacteria* in comparison to the control group (P = 0.001). However, there was no statistically significant difference between PD patients, and control cases as regards *Lactobacilli* (Table 1; Fig. 1).

**Table 1:** Gut Microbiome Results of the PD and the Control Groups

Gut microbiome	Cases (n = 30)	Control (n = 35)	Test of Sig.	p
***Bacteroides***				
Min. – Max.	5.78E-03–9.47E-01	2.25E-03–9.08E-01	U= 355.500	**0.026** ^*^
Mean ± SD.	2.76E-01 ± 2.61E-01	1.48E-01 ± 1.89E-01		
Median	**1.89E-01**	**8.18E-02**		
IQR	5.00E-02–4.83E-01	3.16E-02–1.76E-01		
***Prevotella***				
Min. – Max.	4.33E-06–7.61E-01	1.14E-05–9.00E-01	U = 463.0	0.415
Mean ± SD.	1.92E-01 ± 2.60E-01	1.77E-01 ± 2.42E-01		
Median	2.69E-02	6.95E-02		
IQR	4.30E-04–4.27E-01	5.17E-03–2.44E-01		
***Ruminococcus***				
Min. – Max.	1.39E-04–3.19E-01	1.87E-04–6.34E-01	U = 394.500	0.086
Mean ± SD.	4.78E-02 ± 7.24E-02	7.70E-02 ± 1.14E-01		
Median	2.41E-02	4.82E-02		
IQR	6.75E-03–4.67E-02	1.99E-02–8.55E-02		
***Firmicutes***				
Min. – Max.	3.24E-06–9.52E-01	1.67E-01–9.47E-01	t = 3.331^*^	**0.001** ^*^
Mean ± SD.	3.45E-01 ± 2.25E-01	5.27E-01 ± 2.15E-01		
Median	**3.13E-01**	**5.00E-01**		
IQR	1.82E-01–4.77E-01	4.07E-01–6.84E-01		
***Bacteroidetes***				
Min. – Max.	1.72E-02–8.16E-01	4.17E-02–8.65E-01	t = 0.487	0.628
Mean ± SD.	4.44E-01 ± 2.06E-01	4.19E-01 ± 2.05E-01		
Median	4.44E-01	3.77E-01		
IQR	3.27E-01–5.95E-01	2.86E-01–5.75E-01		
***Lactobacilli***				
Min. – Max.	1.97E-05–5.91E-02	1.55E-05–3.15E-01	U = 424.0	0.184
Mean ± SD.	9.13E-03 ± 1.30E-02	1.61E-02 ± 5.37E-02		
Median	5.26E-03	9.28E-04		
IQR	7.81E-04–1.22E-02	2.50E-04–8.90E-03		
***Bifidobacteria***				
Min. – Max.	8.76E-06–4.90E-02	2.03E-05–1.47E-01	U = 279.0^*^	**0.001** ^*^
Mean ± SD.	8.77E-03 ± 1.26E-02	2.69E-02 ± 3.04E-02		
Median	**1.86E-03**	**2.06E-02**		
IQR	3.63E-04–1.25E-02	4.74E-03–3.42E-02		
**P/B Ratio**				
Min. – Max.	0.0–88.58	0.0–95.97	U = 406.0	0.117
Mean ± SD.	6.24 ± 16.77	8.40 ± 18.78		
Median	0.08	0.65		
IQR	0.0–4.13	0.06–5.24		
**F/B ratio**				
Min. – Max.	0.0–5.65	0.30–17.12	U = 298.0^*^	**0.003** ^*^
Mean ± SD.	1.04 ± 1.16	1.95 ± 2.81		
Median	**0.62**	**1.32**		
IQR	0.34–1.39	0.86–1.91		

t: Student t-test U: Mann Whitney test.

p: p value for association between different categories.

*: Statistically significant at p ≤ 0.05.

### Clinical phenotypes

**Fig. 2:**
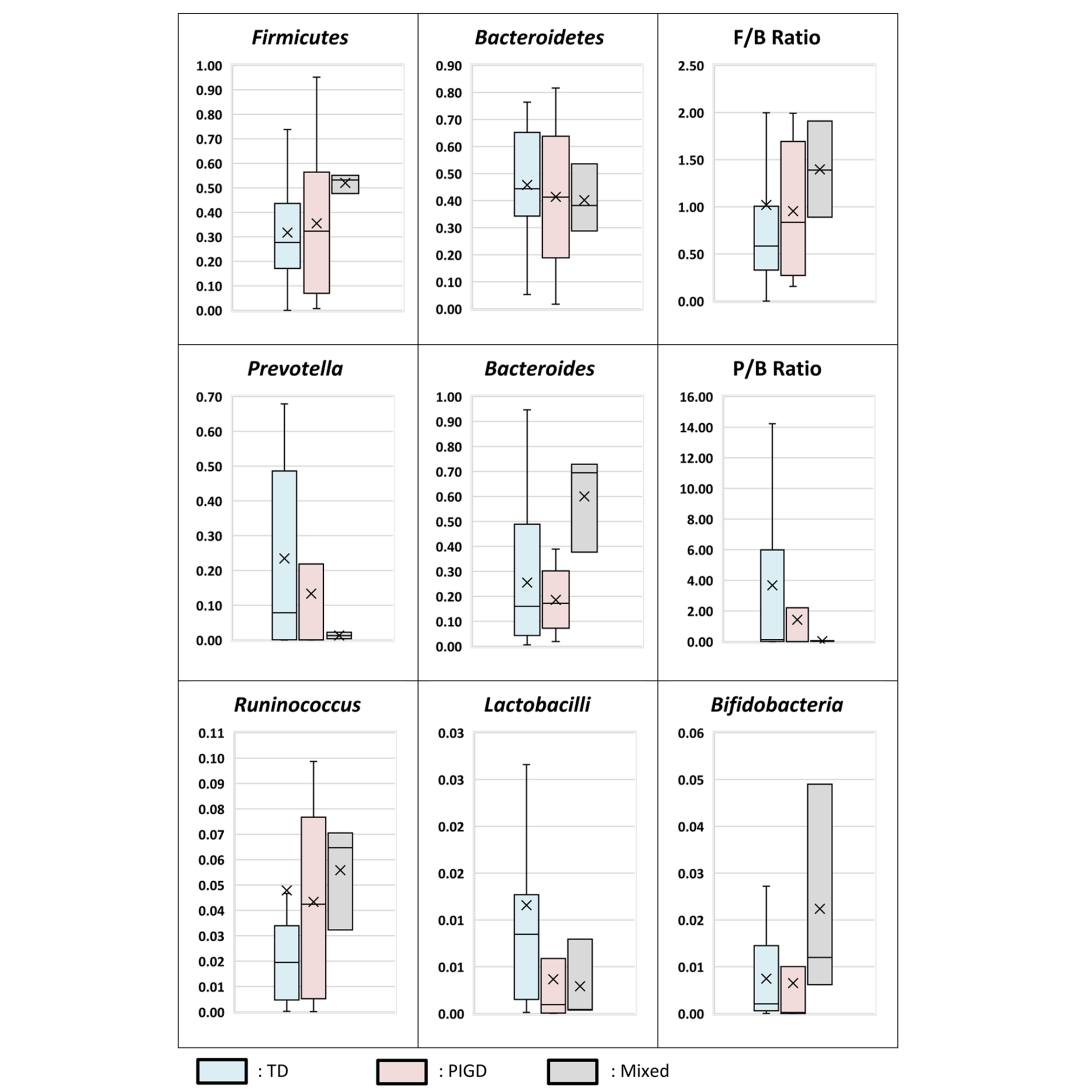
Gut Microbiome Profile of PD Clinical Phenotypes

As regards the predominant phenotypes, the mixed phenotype had significantly higher *Bacteroides*, tremors predominant had lower *Firmicutes* and F/B ratio, and both tremors and PIGD had lower *Bifidobacteria* than the control group. However, there was no statistically significant difference between the 3 PD phenotypes as regards different bacteria and ratios (Table 2; Fig. 2). Moreover, when comparing tremors and non-tremors predominant phenotypes; *Lactobacilli* showed a significant decrease in non-tremors predominant phenotypes (p = 0.025) (Table S2).

**Table 2:** Gut Microbiome Results of the PD Phenotypes and the Control Group

Median Relative Abundance	Control (n = 35)	Phenotype			p
		Tremors (n = 21)	PIGD (n = 6)	Mixed (n = 3)	
***Bacteroides***	**8.18E-02**	1.60E-01	1.72E-01	**6.95E-01**	**0.035** ^*^
**p**_**control**_		0.110	0.328	**0.008** ^*^	
**Sig. bet.**		p_1_ = 0.985, p_2_ = 0.061, p_3_ = 0.100			
***Prevotella***	6.95E-02	7.85E-02	8.65E-04	1.27E-02	0.270
***Ruminococcus***	4.82E-02	1.95E-02	4.25E-02	6.47E-02	
***Firmicutes***	**5.00E-01**	**2.77E-01**	3.23E-01	5.32E-01	**0.007** ^*^
**p**_**control**_		**0.005** ^*^	0.295	1.000	
**Sig. bet.**		p_1_ = 0.982, p_2_ = 0.443, p_3_ = 0.712			
***Bacteroidetes***	3.77E-01	4.44E-01	4.13E-01	3.82E-01	0.902
***Lactobacilli***	9.28E-04	8.47E-03	9.55E-04	4.36E-04	0.100
***Bifidobacteria***	**2.06E-02**	**2.08E-03**	**2.36E-04**	1.20E-02	**0.003** ^*^
**p**_**control**_		**0.004** ^*^	**0.004** ^*^	0.955	
**Sig. bet.**		p_1_ = 0.301, p_2_ = 0.181, p_3_ = 0.065			
**P/B Ratio**	0.65	0.16	0.0	0.02	0.087
**F/B ratio**	**1.32**	**0.58**	0.84	1.39	**0.010** ^*^
**p**_**control**_		**0.002** ^*^	0.120	0.917	
**Sig. bet.**		p_1_ = 0.685, p_2_ = 0.129, p_3_ = 0.289			

p: p-value for the association between Control group and different phenotypes.

p_1_: p-value for association between **Tremors** and **PIGD**.

p_2_: p-value for association between **Tremors** and **Mixed**.

p_3_: p-value for the association between **PIGD** and **Mixed**.

*: Statistically significant at p ≤ 0.05.

### Disease severity

When subgrouping PD patients according to modified H & Y Score; ≤2.5 and > 2.5. There was no statistically significant difference between the 2 severity groups as regards different bacteria and ratios (Table S3).

#### Disease duration

About the relation between the disease duration of PD and the gut microbiome, there was no statistically significant difference between those having the disease for more than 10 years and those less; except for the P/B ratio that was significantly higher in those with PD duration less than 10 years (P = 0.026) (Tables S4).

### Wexner constipation score

There was no statistically significant difference between the gut microbiome and ratios in different constipation severity groups except for; the mild constipation group of PD cases had significantly lower *Firmicutes* (p = 0.041) and F/B ratio (p = 0.026). Also, the mild constipation severity group of PD cases had higher *Prevotella*, P/B ratio, and *Bifidobacteria*, however, the difference was not statistically significant (Table S5).

### Correlation with clinical parameters

When studying the correlation between the different gut microbiota relative abundance and the disease duration as well as Wexner Score and severity (Motor symptoms, NMS score, and UPDRS), no statistically significant correlation was detected except for a significant positive correlation between the disease duration and *Ruminococcus* relative abundance (r = 0.378, p = 0.035) (Table S6).

### Alpha diversity

Shannon diversity index, which considers both species richness and evenness, demonstrated a statistically non-significant lower microbial diversity in the PD group (Mean DI = 1.22) compared to the control group (1.25). No statistically significant difference was observed between the three phenotypes: Tremors (1.24), PIGD (1.15), and Mixed (1.28) predominant phenotypes.

### Bray-Curtis dissimilarity index

The Bray-Curtis dissimilarity index was performed to study the dissimilarity between both the PD cases and the healthy control subjects. The median dissimilarity index between PD cases and healthy controls was 35.5%. Comparing the dissimilarity index between Tremors predominant cases (37%), PIGD (32%), and Mixed phenotypes (28%), demonstrated no statistically significant difference.

## Discussion

Our results revealed that PD cases have gut bacterial dysbiosis with a 35.5% dissimilarity index compared to the control subjects. Using the F/B ratio as a signature of gut dysbiosis; the F/B ratio of PD patients (0.62) was significantly lower than the healthy control (1.32). These findings agreed with many studies which have suggested the potential relationship between the change in the composition of the gut microbiota and PD [11, 12].

The present study showed a significant decrease of *Firmicutes* and an insignificant increase of *Bacteroidetes* in PD patients. This is consistent with previous studies by Keshavarzian et al. and Lin et al. who showed a significant decrease in *Firmicutes* in their patients [11, 12]. In contrast, Li et al. in China found a significant decrease in *Bacteroidetes* phylum and that *Firmicutes* was nearly the same in patients and controls [17]. Moreover, Unger et al. and Bedarf et al. declared a mild increase in *Firmicutes* in PD cases [18, 19].

The mild increase in *Firmicutes* in the study of Bedarf et al., can be explained by the fact that all the PD study participants were male and none of them had gastrointestinal complaints including constipation whereas all our PD cases had constipation [19]. However, the strongest evidence is with the decrease of *Firmicutes* in Parkinson’s disease patients, for the important role of *Firmicutes* in the production of butyrate, which aids in the maintenance of the gut barrier [8]. Therefore, it is reasonable to expect *Firmicutes* to be decreased in the gut of PD patients.

The present study revealed a significant increase in the proinflammatory *Bacteriodes* among PD patients. However, *Prevotella* and P/B ratio was decreased in PD patients, but the difference was not statistically significant. Of note, the P/B ratio was significantly lower in those with PD duration more than 10 years.

Indeed, most previous studies have shown decreased *Prevotella* in the gut of PD patients. Our results showed that this decrease was not statistically significant as many studies like those designed by Keshavarzian et al. in the USA, Li et al. in China, Unger et al. in Germany and Hasegawa et al. in Japan who have demonstrated a non-significant decrease of *Prevotella* in PD patients [11, 17, 18, 20].

On the other hand, Scheperjans et al. in Finland, Bedarf et al. in Germany and Petrov et al. in Russia, have declared a significant reduction in *Prevotella* species in their PD patients [10, 19, 21]. Surprisingly, Hill-Burn et al., as well as Barichella et al., failed to find an association between *Prevotella* and Parkinson’s disease in the 2 large studies performed in USA 2017 and Italy 2018 respectively [22, 23]. This contrasts with the study done by Keshavarzian et al. in the USA and the study performed by Tetz et al. also among the American population and which revealed a depletion of *Prevotellaceae* abundance in the PD samples [11, 24].

Unexpectedly, Heintz-Buschart et al. demonstrated a significantly increased abundance of *Prevotella* in the gut microbiota of PD patients compared with healthy controls. This strange finding may be attributed to factors in the study population, methods used in the analysis, or other factors. Patients’ samples were collected over about 3 years and were preserved; this long period might have an impact on the results. Indeed, a lot of results in this study are incompatible with the vast majority of other studies; for example, they found increased *Prevotella* in PD samples; a finding which is, to our knowledge, unique to the above-mentioned studies [25].

Although *Prevotella* and *Bacteroides* are both mucin degraders, their relative percentage can affect the rate and result of this process. The process of mucin degradation and secretion should be in equilibrium to keep integrity of gut barrier and provide nutrients for gut microbiota. Increased the proportion of *Bacteriodes* in relation to *Prevotella* may affect the gut barrier and provoke inflammation. In our study, we found a significant increase of *Bacteriodes* in fecal samples of PD patients. This trend of increased *Bacteriodes* and decreased *Prevotella* is consistent with the disintegrity of gut barrier expected in Parkinson’s Disease, and this agrees with multiple studies and disagrees with others [8, 26].

Keshavarzian et al. found a significant increase of *Bacteriodes* in his PD samples [11]. Also, Lin et al. in studying patients with Parkinson’s disease in southern China, have found a significant increase in *Bacteroides* [12]. Moreover, Hopfner et al. demonstrated an insignificant increase in *Bacteroides* in PD patients in their study done in Germany [27].

For *Ruminococcus*, we found an insignificant decreased level in samples of our PD patients compared to controls. However, its level was positively associated with disease duration and severe motor phenotypes (Mixed and PIGD). Indeed, the results of previous studies as regards *Ruminococcus* are contradictory. Some declared increased *Ruminococcus* in the gut of their PD patients [22, 25], while others found decreased *Ruminococcus* in samples of PD subjects compared to controls [19, 28]. It seems that *Ruminococcus* may be decreased in PD subjects at the beginning of the disease and can constitute a risk; and as time passes, the severity of disease increases and the relative abundance of this genus can be increased in some subjects like that observed in our patients. There are some observations that can support our assumption. For example, Barichella et al. have found a decreased abundance of *Ruminococcus* in PD samples in their large study that included a large number of early-stage PD patients (57 patients) [23]. Also, this genus was reduced in the study performed by Bedarf et al. among 31 early-stage levodopa-naïve patients, when compared with 28 healthy controls [19].

As regards *Lactobacillus*, we noticed an insignificant increased relative abundance in PD patients compared to controls. We also found a significant decrease of *Lactobacillus* in non-tremor phenotypes; that needs further studies to see if there is a certain mechanism through which this bacteria can affect this feature.

*Lactobacillus* relative abundance was noticed to be increased in PD patients in several studies [10, 20–23, 27], while being decreased in a fewer number of studies [18, 19, 28, 29]. The increased abundance of *Lactobacilli* has been linked to decreased ghrelin concentration which helps protect and maintain normal striatal dopamine function [21].

About *Bifidobacterium*, it was significantly reduced in our PD cases compared to controls; this disagrees with most previous studies. We also found a significant decreased levels in tremor dominant and PIGD subgroups compared to controls. In the case of *Bifidobacterium*, most studies revealed an increased abundance of this genus [10, 21, 23, 24]. However, in accordance with our study, Tan et al. in a Malaysian study found that PD diagnosis was significantly associated with decreased abundance of *Bifidobacterium* [30]. We assume that low *Bifidobacterium* level is risky for PD and its progression, and that increased level observed in most studies appears to be rather a compensatory mechanism which may take place at different stages and duration of the disease and may occur more in severe stages, like in our study.

As regards the relation between gut microbiota and clinical phenotypes in our study; as expected the mixed phenotype had significantly higher the proinflammatory *Bacteroides*, tremor predominant had lower *Firmicutes* and F/B ratio, and both tremors and PIGD had lower *Bifidobacteria* than the control group. However, there was no statistically significant difference between the three PD phenotypes as regards different bacteria and ratios. This is in accordance with some other studies [11, 19].

Additionally, we did not find a significant relation between most microbiota and the clinical phenotypes of PD (Tremor, PIGD & Mixed) and the severity groups; complying with previous studies [10, 11, 18]. When we combined PIGD and Mixed phenotypes in comparison with the Tremors phenotype, all results were not significant except for decreased *Lactobacilli* in combined PIGD and Mixed phenotype, in contrast to the results reported by Barichella et al. [23].

According to Wexner Score, 70% of our PD cases versus 30% of the control cases had moderate to very severe constipation. There was a statistically significant difference between the two groups as regards total Wexner Constipation Score which was more severe in PD patients. Also, all the variables were statistically significant more severe in PD cases than control, except for frequency of bowel movement and duration of constipation.

As regards the gut microbiome, the mild constipation severity group of PD cases had higher *Prevotella*, P/B ratio and *Bifidobacteria* indicating better condition of gut barrier, however the difference was not statistically significant which could be explained by the small number of mild cases in our PD study group. This small number of mild cases also had significantly lower *Firmicutes* and lower F/B ratio which we could not explain.

Decreased diversity of gut microbiota has been associated with intestinal inflammation which can trigger misfolding of α-synuclein in GI neurons [20]. Evaluation of gut microbiota in our PD patients showed an insignificant decreased level of alpha diversity compared to controls. Dissimilarities have been demonstrated in different studies as regards the presence of significant differences in diversity between patients and control groups. For example, Scheperjans and his colleagues as well as Hopfner et al. have found an insignificant difference in the alpha diversity between PD cases and controls [10, 27]. However, Petrov et al. have found a significant decrease in alpha diversity in PD patients which is different from our results as they used sequencing technique detecting more bacteria than Real time PCR applied in the present research [21].

### Limitations

The limitation in our study was the small sample size that can probably lead to loss of statistical significance at certain points.

#### Conclusion

The current study showed an evidence of changes in the gut microbiome of PD patients compared to the healthy controls. These observations may highlight the importance of identification of microbiome and specific bacterial changes that can be targeted for treatment of PD.

## Materials and methods

### Patients

The present study was conducted on 30 PD cases, who presented to the PD Neurology Clinic of Alexandria University Hospital. Thirty-five age- and sex-matched healthy subjects, without any signs of PD or potential premotor symptoms, were included as the control group.

We included patients of age above 60 years old with a diagnosis of PD based on the Queen Square Brain Bank criteria [31].

Our exclusion criteria were chosen to cover several conditions that can independently affect the gut microbiota. These included severe renal or liver impairment, inflammatory bowel disease, patients with other neurological disorders, and a history of recent antibiotic use.

### History & clinical data

A detailed history and full clinical examination were performed for patients and control subjects.

Parkinsonian symptoms and severity were measured using the Unified Parkinson’s Disease Rating Scale (UPDRS) and the modified Hoehn & Yahr scale (H&Y) [32, 33]. Overall NMS severity was assessed using the Non-Motor Symptoms Scale (NMSS) [34]. The PD cases were clinically classified into 3 phenotypes; tremor dominant (TD), postural instability and gait difficulty (PIGD), and mixed phenotypes (MX) as described by Jankovic et al. [32]. Tremor-dominant PD was considered if the mean tremor score/mean PIGD score in (UPDRS) was greater than or equal to 1.5, while the PIGD-dominant type if the mean tremor score/mean PIGD score was less than or equal to 1.0; with values in between described as mixed type [32]. Patients with H&Y stage ranging from 1 − 2.5 were included in the mild PD (MPD) group, while patients with H&Y stage ranging from 3 − 5 were included in the severe PD (SPD) group [33]. The degree of constipation was quantified in more detail using the Wexner constipation score (Cleveland Clinic Constipation Scoring System) [35].

### Gut microbiome analysis

#### Specimen collection, preservation, and transport

Following defecation at home in sterile containers, stool samples from patients and controls were maintained in the freezer, transported to Alexandria University-Main Microbiology laboratory frozen, and then kept at -80 °C for further processing.

#### DNA extraction

DNA was extracted from 220 mg stool samples using QIAamp DNA Stool Extraction Mini Kit (Qiagen, Germany) in accordance with the manufacturer’s instructions. The extracted DNA was kept at -80 °C until PCR analysis [36].

#### SYBR green real-time PCR

##### Primers

Oligonucleotide primers targeted the 16 S ribosomal RNA (rRNA) gene sequences of *Bacteroides, Prevotella, Ruminococcus, Firmicutes, Bacteroidetes, Lactobacilli*, and *Bifidobacteria* were used. Primers were also used to amplify a conserved 16 S rDNA sequence present in all bacteria (universal primer set, recognizing domain bacteria), the amplification of which served as the denominator against which the amplification of the other bacteria was compared. All of the primers (Invitrogen, USA) were described from previously published study [36].

##### Detection and quantitation

Amplification was performed in a light cycler [Rotor Gene Q, Qiagen, Germany] using a SensiFAST TM SYBR No-ROX PCR kit (Bioline Co. UK). In short, forward and reverse primers (4 pmol each) were used in 20 µl reactions containing 2 µl of the DNA extract. PCR amplification was performed with initial denaturation at 95 ˚C for 10 min, followed by 40 cycles of denaturation at 95 ˚C for 30 s, annealing at 60 ˚C for 30 s, and extension at 72 ˚C for 30 s. Melting curve analysis was performed to check the specificity of the amplified products. The relative quantitation is calculated automatically by the Rotor-Gene software and expressed as a relative fold difference [36].

#### Statistical analysis of the data

Data of the results were analyzed using IBM SPSS software package version 20.0 (Armonk, NY: IBM Corp). Qualitative data were described using number and percent. The Shapiro-Wilk test was used to verify the normality of distribution Quantitative data were described using range (minimum and maximum), mean, standard deviation, median and interquartile range (IQR). The Chi-Square, Fisher’s Exact, and Monte Carlo tests were used for qualitative variables and Mann-Whitney, Kruskal Wallis tests for quantitative ones. Spearman Correlation Coefficient was calculated for assessing correlations between different quantitative variables. Significance of the obtained results was judged at the 5% level.

To assess the degree of variation of the microbial community structure within a sample, we measured the alpha diversity by employing the Shannon diversity index [37], and to evaluate the degree of similarity between PD cases and the control group we employed the Bray-Curtis similarity index [38].

### Electronic supplementary material

Below is the link to the electronic supplementary material.

Supplementary Material 1

## Data Availability

All data will be available from the corresponding author on reasonable request.

## List of abbreviations

TD: Tremors Dominant
PIGD: Postural instability and gait difficulty
MX: Mixed phenotype
ENS: Enteric Nervous System
PD: Parkinson’s Disease
