# Correction: Study of the gut microbiome in Egyptian patients with Parkinson’s Disease

**DOI:** 10.1186/s12866-024-03404-3

**Published:** 2024-07-01

**Authors:** Mohammad Mehanna, Suzan Abou-Raya, Shwikar Mahmoud Ahmed, Ghada Ashmawy, Ahmed Ibrahim, Essameldin AbdelKhaliq

**Affiliations:** 1 Internal Medicine, Faculty of Medicine, University of Alexandria, Alexandria, Egypt; 2 Medical Microbiology and Immunology Department, Faculty of Medicine, University of Alexandria, Alexandria, Egypt; 3 Department of Neuropsychiatry, Faculty of Medicine, University of Alexandria, Alexandria, Egypt

**Correction:** ***BMC Microbiol*** **23, 196 (2023)**

10.1186/s12866-023-02933-7

Following the publication of the original article [1], the authors would like to change the second author’s name from “Suzan AbuRaya” to “Suzan Abou-Raya”. This has been corrected in the title as well as in the Author’s Affiliation section.

## References

[1] Mehanna, M., Abou-Raya, S., Ahmed, S.M. *et al* Study of the gut microbiome in Egyptian patients with Parkinson’s Disease. *BMC Microbiol* **23**, 196 (2023). 10.1186/s12866-023-02933-7 10.1186/s12866-023-02933-7 PMC10362707 37481569

