# Supplementary material for: Study of the gut microbiome in Egyptian patients with Parkinson’s Disease
Source: BMC Microbiol. 2023 Jul 22;23:196. doi: 10.1186/s12866-023-02933-7 (PMC10362707; doi:10.1186/s12866-023-02933-7)
Supplement: Supplementary file 1 — Supplementary Material 1 [file 12866_2023_2933_MOESM1_ESM.docx]

**Table (S 1):** Wexner Constipation Score for PD cases and Controls

| **Wexner Constipation Score** | **Cases (n = 30)** | **Control (n = 35)** | **U** | **p** |
| --- | --- | --- | --- | --- |
| **Defecation times** |  |  |  |  |
| Min. – Max. | 0.0 – 4.0 | 0.0 – 4.0 | 463.50 | 0.384 |
| Median (IQR) | 1.0 (0.0 – 2.0) | 0.0 (0.0 – 1.50) |  |  |
| **Defecation difficulty** |  |  |  |  |
| Min. – Max. | 0.0 – 4.0 | 0.0 – 4.0 | 219.0^*^ | **<0.001^*^** |
| Median (IQR) | 2.0 (1.0 – 4.0) | 0.0 (0.0 – 1.0) |  |  |
| **Sense of incomplete evacuation** |  |  |  |  |
| Min. – Max. | 0.0 – 4.0 | 0.0 – 4.0 | 276.50^*^ | **0.001^*^** |
| Median (IQR) | 1.0 (1.0 – 3.0) | 0.0 (0.0 – 1.0) |  |  |
| **Abdominal pain** |  |  |  |  |
| Min. – Max. | 0.0 – 3.0 | 0.0 – 2.0 | 309.50^*^ | **0.002^*^** |
| Median (IQR) | 1.0 (1.0 – 2.0) | 0.0 (0.0 – 1.0) |  |  |
| **Minutes in lavatory per attempt** |  |  |  |  |
| Min. – Max. | 0.0 – 4.0 | 0.0 – 2.0 | 162.50^*^ | **<0.001^*^** |
| Median (IQR) | 1.0 (0.0 – 3.0) | 0.0 (0.0 – 0.0) |  |  |
| **Need for help** |  |  |  |  |
| Min. – Max. | 0.0 – 2.0 | 0.0 – 2.0 | 252.0^*^ | **<0.001^*^** |
| Median (IQR) | 1.0 (0.0 – 2.0) | 0.0 (0.0 – 0.0) |  |  |
| **Times of failed defecation** |  |  |  |  |
| Min. – Max. | 0.0 – 2.0 | 0.0 – 2.0 | 273.0^*^ | **<0.001^*^** |
| Median (IQR) | 1.0 (1.0 – 1.0) | 0.0 (0.0 – 0.0) |  |  |
| **Duration of constipation (years)** |  |  |  |  |
| Min. – Max. | 0.0 – 3.0 | 0.0 – 16.0 | 451.0 | 0.315 |
| Median (IQR) | 1.0 (0.0 – 2.0) | 1.0 (0.0 – 6.0) |  |  |
| **Total score** |  |  |  |  |
| Min. – Max. | 1.0 – 18.0 | 0.0 – 16.0 | 166.5^*^ | **<0.001^*^** |
| Median (IQR) | 12.50 (5.0 – 16.0) | 1.0 (0.0 – 6.0) |  |  |

**U: Mann Whitney test**

p: p value for comparing between the studied groups

*: Statistically significant at p ≤ 0.05

**Table (S 2):** Relation between tremors and non-tremors phenotypes and Gut microbiome

| **Gut microbiome** | **Phenotype** | | **Test of Sig.** | **p** |
| --- | --- | --- | --- | --- |
|  | **Tremors (n = 21)** | **Mixed +PIGD (n = 9)** |  |  |
| ***Bacteroides*** |  |  |  |  |
| Min. – Max. | 5.78E-03 – 9.47E-01 | 1.92E-02 – 7.29E-01 | U= 74.0 | 0.372 |
| Mean ± SD. | 2.55E-01 ± 2.67E-01 | 3.24E-01 ± 2.54E-01 |  |  |
| Median | **1.60E-01** | **2.73E-01** |  |  |
| ***Prevotella*** |  |  |  |  |
| Min. – Max. | 2.98E-05 – 6.79E-01 | 4.33E-06 – 7.61E-01 | U= 61.0 | 0.137 |
| Mean ± SD. | 2.35E-01 ± 2.58E-01 | 9.33E-02 ± 2.51E-01 |  |  |
| Median | **7.85E-02** | **3.77E-03** |  |  |
| ***Ruminococcus*** |  |  |  |  |
| Min. – Max. | 2.41E-04 – 3.19E-01 | 1.39E-04 – 9.87E-02 | U= 60.0 | 0.125 |
| Mean ± SD. | 4.79E-02 ± 8.48E-02 | 4.75E-02 ± 3.18E-02 |  |  |
| Median | **1.95E-02** | **4.30E-02** |  |  |
| ***Firmicutes*** |  |  |  |  |
| Min. – Max. | 3.24E-06 – 7.38E-01 | 7.23E-03 – 9.52E-01 | t= 1.037 | 0.308 |
| Mean ± SD. | 3.17E-01 ± 1.99E-01 | 4.10E-01 ± 2.79E-01 |  |  |
| Median | **2.77E-01** | **4.35E-01** |  |  |
| ***Bacteroidetes*** |  |  |  |  |
| Min. – Max. | 5.31E-02 – 7.64E-01 | 1.72E-02 – 8.16E-01 | t= 0.577 | 0.568 |
| Mean ± SD. | 4.58E-01 ± 2.00E-01 | 4.10E-01 ± 2.28E-01 |  |  |
| Median | **4.44E-01** | **3.82E-01** |  |  |
| ***Lactobacilli*** |  |  |  | **0.025^*^** |
| Min. – Max. | 1.25E-04 – 5.91E-02 | 1.97E-05 – 1.83E-02 | U= 45.0^*^ |  |
| Mean ± SD. | 1.16E-02 ± 1.45E-02 | 3.42E-03 ± 6.11E-03 |  |  |
| Median | **8.47E-03** | **4.36E-04** |  |  |
| ***Bifidobacteria*** |  |  |  |  |
| Min. – Max. | 1.36E-05 – 2.72E-02 | 8.76E-06 – 4.90E-02 | U= 83.0 | 0.625 |
| Mean ± SD. | 7.47E-03 ± 9.22E-03 | 1.18E-02 ± 1.85E-02 |  |  |
| Median | **2.08E-03** | **8.06E-04** |  |  |
| **P/B Ratio** |  |  |  |  |
| Min. – Max. | 0.0 – 88.58 | 0.0 – 8.43 | U= 52.0 | 0.056 |
| Mean ± SD. | 8.50 ± 19.67 | 0.96 ± 2.80 |  |  |
| Median | **0.16** | **0.01** |  |  |
| **F/B Ratio** |  |  |  |  |
| Min. – Max. | 0.0 – 5.65 | 0.16 – 1.99 | U= 72.0 | 0.326 |
| Mean ± SD. | 1.02 ± 1.33 | 1.10 ± 0.69 |  |  |
| Median | **0.58** | **1.25** |  |  |
| **Diversity index** |  |  |  |  |
| Min. – Max. | 0.97 – 1.46 | 0.91 – 1.41 | t= 0.669 | 0.509 |
| Mean ± SD. | 1.24 ± 0.15 | 1.20 ± 0.16 |  |  |
| Median | **1.22** | **1.23** |  |  |
| **Dissimilarity index (%)** |  |  |  |  |
| Min. – Max. | 21.0 – 66.0 | 22.0 – 92.0 | U= 65.50 | 0.193 |
| Mean ± SD. | 38.71 ± 11.15 | 37.44 ± 22.37 |  |  |
| Median | **37.0** | **30.0** |  |  |

t: Student t-test U: Mann Whitney test

p: p value for association between different categories

*: Statistically significant at p ≤ 0.05

**Table (S 3):** Relation between Hoehn and Yahr Severity Score and the Gut microbiome

| **Gut microbiome** | **Hoehn and Yahr Severity Score** | | **Test of sig.** | **p** |
| --- | --- | --- | --- | --- |
|  | **≤2.5 (n = 25)** | **>2.5 (n = 5)** |  |  |
| ***Bacteroides*** |  |  |  |  |
| Min. – Max. | 5.78E-03 – 9.47E-01 | 9.03E-02 – 7.29E-01 | U= 35.0 | 0.136 |
| Mean ± SD. | 2.45E-01 ± 2.52E-01 | 4.29E-01 ± 2.78E-01 |  |  |
| Median | 1.60E-01 | 3.77E-01 |  |  |
| ***Prevotella*** |  |  |  |  |
| Min. – Max. | 4.33E-06 – 6.79E-01 | 3.09E-04 – 7.61E-01 | U= 59.0 | 0.872 |
| Mean ± SD. | 1.99E-01 ± 2.51E-01 | 1.60E-01 ± 3.36E-01 |  |  |
| Median | 3.73E-02 | 1.27E-02 |  |  |
| ***Ruminococcus*** |  |  |  |  |
| Min. – Max. | 1.39E-04 – 3.19E-01 | 6.91E-03 – 9.87E-02 | U= 35.0 | 0.136 |
| Mean ± SD. | 4.64E-02 ± 7.82E-02 | 5.46E-02 ± 3.56E-02 |  |  |
| Median | 2.30E-02 | 6.47E-02 |  |  |
| ***Firmicutes*** |  |  |  |  |
| Min. – Max. | 3.24E-06 – 7.38E-01 | 9.03E-02 – 9.52E-01 | t= 2.007 | 0.054 |
| Mean ± SD. | 3.10E-01 ± 1.95E-01 | 5.20E-01 ± 3.06E-01 |  |  |
| Median | 2.77E-01 | 5.32E-01 |  |  |
| ***Bacteroidetes*** |  |  |  |  |
| Min. – Max. | 1.72E-02 – 8.16E-01 | 2.88E-01 – 5.79E-01 | t=  0.105 | 0.917 |
| Mean ± SD. | 4.42E-01 ± 2.21E-01 | 4.53E-01 ± 1.18E-01 |  |  |
| Median | 4.43E-01 | 4.78E-01 |  |  |
| ***Lactobacilli*** |  |  |  |  |
| Min. – Max. | 1.97E-05 – 5.91E-02 | 3.70E-04 – 7.95E-03 | U= 39.0 | 0.208 |
| Mean ± SD. | 1.05E-02 ± 1.38E-02 | 2.40E-03 ± 3.16E-03 |  |  |
| Median | 7.12E-03 | 1.50E-03 |  |  |
| ***Bifidobacteria*** |  |  |  |  |
| Min. – Max. | 8.76E-06 – 3.77E-02 | 1.10E-04 – 4.90E-02 | U= 56.0 | 0.746 |
| Mean ± SD. | 7.81E-03 ± 1.07E-02 | 1.35E-02 ± 2.04E-02 |  |  |
| Median | 1.64E-03 | 6.17E-03 |  |  |
| ***P/B Ratio*** |  |  |  |  |
| Min. – Max. | 0.0 – 88.58 | 0.0 – 8.43 | U= 49.0 | 0.481 |
| Mean ± SD. | 7.15 ± 18.23 | 1.70 ± 3.76 |  |  |
| Median | 0.14 | 0.02 |  |  |
| ***F/B ratio*** |  |  |  |  |
| Min. – Max. | 0.0 – 5.65 | 0.16 – 1.99 | U= 41.0 | 0.251 |
| Mean ± SD. | 1.0 ± 1.23 | 1.27 ± 0.76 |  |  |
| Median | 0.58 | 1.39 |  |  |
| **Diversity Index** |  |  |  |  |
| Min. – Max. | 0.91 – 1.46 | 1.08 – 1.31 | t=  0.191 | 0.850 |
| Mean ± SD. | 1.23 ± 0.16 | 1.21 ± 0.10 |  |  |
| Median | 1.22 | 1.23 |  |  |
| **Dissimilarity index (%)** |  |  |  |  |
| Min. – Max. | 21.0 – 92.0 | 22.0 – 52.0 | U= 42.50 | 0.275 |
| Mean ± SD. | 39.44 ± 15.55 | 32.80 ± 11.37 |  |  |
| Median | 36.0 | 30.0 |  |  |

t: Student t-test U: Mann Whitney test

p: p value for association between different categories

*: Statistically significant at p ≤ 0.05

**Table (S 4):** Relation between the disease duration and the gut microbiome

| **Gut microbiome** | **Disease duration** | | **Test of Sig.** | **p** |
| --- | --- | --- | --- | --- |
|  | **<10 (n = 19)** | **≥10 (n = 11)** |  |  |
| ***Bacteroides*** |  |  |  |  |
| Min. – Max. | 5.78E-03 – 7.29E-01 | 1.92E-02 – 9.47E-01 | U= 78.0 | 0.268 |
| Mean ± SD. | 2.38E-01 ± 2.47E-01 | 3.40E-01 ± 2.83E-01 |  |  |
| Median | **1.60E-01** | **2.73E-01** |  |  |
| ***Prevotella*** |  |  |  |  |
| Min. – Max. | 2.98E-05 – 6.79E-01 | 4.33E-06 – 7.61E-01 | U= 61.0 | 0.064 |
| Mean ± SD. | 2.55E-01 ± 2.63E-01 | 8.33E-02 ± 2.26E-01 |  |  |
| Median | **9.89E-02** | **1.42E-03** |  |  |
| ***Ruminococcus*** |  |  |  |  |
| Min. – Max. | 2.41E-04 – 2.45E-01 | 1.39E-04 – 3.19E-01 | U= 68.0 | 0.123 |
| Mean ± SD. | 3.70E-02 ± 6.06E-02 | 6.64E-02 ± 8.93E-02 |  |  |
| Median | **1.95E-02** | **4.30E-02** |  |  |
| ***Firmicutes*** |  |  |  |  |
| Min. – Max. | 6.67E-02 – 7.38E-01 | 3.24E-06 – 9.52E-01 | t= 0.412 | 0.683 |
| Mean ± SD. | 3.58E-01 ± 1.93E-01 | 3.23E-01 ± 2.80E-01 |  |  |
| Median | **3.26E-01** | **2.54E-01** |  |  |
| ***Bacteroidetes*** |  |  |  |  |
| Min. – Max. | 5.31E-02 – 7.64E-01 | 1.72E-02 – 8.16E-01 | t= 0.684 | 0.500 |
| Mean ± SD. | 4.63E-01 ± 1.90E-01 | 4.09E-01 ± 2.37E-01 |  |  |
| Median | **4.44E-01** | **4.43E-01** |  |  |
| ***Lactobacilli*** |  |  |  |  |
| Min. – Max. | 1.25E-04 – 5.91E-02 | 1.97E-05 – 1.83E-02 | U= 68.0 | 0.123 |
| Mean ± SD. | 1.19E-02 ± 1.51E-02 | 4.29E-03 ± 6.15E-03 |  |  |
| Median | **8.47E-03** | **1.68E-03** |  |  |
| ***Bifidobacteria*** |  |  |  |  |
| Min. – Max. | 1.36E-05 – 2.72E-02 | 8.76E-06 – 4.90E-02 | U= 94.0 | 0.672 |
| Mean ± SD. | 7.78E-03 ± 9.63E-03 | 1.05E-02 ± 1.69E-02 |  |  |
| Median | **2.08E-03** | **1.44E-03** |  |  |
| ***P/B Ratio*** |  |  |  | **0.026^*^** |
| Min. – Max. | 0.0 – 88.58 | 0.0 – 8.43 | U= 53.0^*^ |  |
| Mean ± SD. | 9.39 ± 20.52 | 0.80 ± 2.53 |  |  |
| Median | **0.62** | **0.01** |  |  |
| ***F/B ratio*** |  |  |  |  |
| Min. – Max. | 0.10 – 5.65 | 0.0 – 1.99 | U= 90.0 | 0.553 |
| Mean ± SD. | 1.16 ± 1.35 | 0.84 ± 0.73 |  |  |
| Median | **0.66** | **0.47** |  |  |
| **Diversity Index** |  |  |  |  |
| Min. – Max. | 0.97 – 1.46 | 0.91 – 1.43 | t = 0.706 | 0.486 |
| Mean ± SD. | 1.24 ± 0.14 | 1.20 ± 0.17 |  |  |
| Median | **1.23** | **1.21** |  |  |
| **Dissimilarity index (%)** |  |  |  |  |
| Min. – Max. | 21.0 – 55.0 | 22.0 – 92.0 | U= 93.50 | 0.641 |
| Mean ± SD. | 35.68 ± 9.07 | 42.91 ± 21.58 |  |  |
| Median | **36.0** | **34.0** |  |  |

t: Student t-test U: Mann Whitney test

p: p value for association between different categories

*: Statistically significant at p ≤ 0.05

**Table (S 5):** Wexner Constipation Score and Gut microbiome in PD cases

| **Gut microbiome** | **Wexner Constipation Score** | | | | **Test of Sig.** | **p** |
| --- | --- | --- | --- | --- | --- | --- |
|  | **Mild (0 – 5) (n = 9)** | **Moderate (6 – 10) (n = 3)** | **Severe (11 – 15) (n = 10)** | **Very severe (16–20) (n = 8)** |  |  |
| ***Bacteroides*** |  |  |  |  |  |  |
| Min. – Max. | 5.78E-03 – 9.47E-01 | 2.26E-01 – 6.96E-01 | 1.92E-02 – 6.95E-01 | 2.20E-02 – 7.29E-01 | H= 1.869 | 0.600 |
| Mean ± SD. | 2.52E-01 ± 3.15E-01 | 4.05E-01 ± 2.54E-01 | 2.68E-01 ± 2.46E-01 | 2.63E-01 ± 2.53E-01 |  |  |
| Median | **1.60E-01** | **2.93E-01** | **1.83E-01** | **1.84E-01** |  |  |
| ***Prevotella*** |  |  |  |  |  |  |
| Min. – Max. | 1.98E-04 – 6.79E-01 | 2.98E-05 – 2.36E-02 | 4.33E-06 – 7.61E-01 | 3.09E-04 – 4.79E-01 | H= 2.931 | 0.402 |
| Mean ± SD. | 2.52E-01 ± 2.69E-01 | 1.13E-02 ± 1.18E-02 | 2.48E-01 ± 3.15E-01 | 1.23E-01 ± 2.03E-01 |  |  |
| Median | **9.89E-02** | **1.02E-02** | **3.02E-02** | **2.60E-03** |  |  |
| ***Ruminococcus*** |  |  |  |  |  |  |
| Min. – Max. | 3.23E-04 – 4.67E-02 | 8.93E-03 – 4.08E-02 | 1.39E-04 – 2.45E-01 | 4.54E-03 – 3.19E-01 | H= 4.823 | 0.185 |
| Mean ± SD. | 1.46E-02 ± 1.55E-02 | 2.44E-02 ± 1.60E-02 | 6.02E-02 ± 7.87E-02 | 7.82E-02 ± 1.02E-01 |  |  |
| Median | **6.75E-03** | **2.36E-02** | **3.71E-02** | **4.59E-02** |  |  |
| ***Firmicutes*** |  |  |  |  |  | **0.041^*^** |
| Min. – Max. | 3.24E-06 – 4.46E-01 | 3.26E-01 – 5.99E-01 | 7.23E-03 – 7.38E-01 | 1.82E-01 – 9.52E-01 | F= 3.169^*^ |  |
| Mean ± SD. | 1.97E-01 ± 1.18E-01 | 5.07E-01 ± 1.56E-01 | 3.34E-01 ± 2.33E-01 | 4.65E-01 ± 2.47E-01 |  |  |
| Median | **1.83E-01** | **5.95E-01** | **3.66E-01** | **4.12E-01** |  |  |
| ***Bacteroidetes***  Min. – Max.  Mean ± SD.  Median | 1.06E-01 – 7.64E-01  4.56E-01 ± 1.96E-01  **4.43E-01** | 1.66E-01 – 4.74E-01  3.44E-01 ± 1.60E-01  **3.93E-01** | 1.72E-02 – 8.16E-01  4.92E-01 ± 2.46E-01  **5.58E-01** | 5.31E-02 – 6.57E-01  4.06E-01 ± 1.94E-01  **3.71E-01** | F= 0.486 | 0.695 |
| ***Lactobacilli***  Min. – Max.  Mean ± SD.  Median | 1.25E-04 – 1.30E-02  6.34E-03 ± 5.38E-03  **7.12E-03** | 7.81E-04 – 2.66E-02  1.20E-02 ± 1.33E-02  **8.47E-03** | 1.97E-05 – 1.31E-02  5.35E-03 ± 5.62E-03  **3.49E-03** | 4.36E-04 – 5.91E-02  1.59E-02 ± 2.21E-02  **3.39E-03** | H= 1.533 | 0.675 |
| ***Bifidobacteria***  Min. – Max.  Mean ± SD.  Median | 1.36E-05 – 2.72E-02  8.84E-03 ± 9.13E-03  **8.87E-03** | 1.79E-05 – 1.65E-02  5.69E-03 ± 9.37E-03  **5.43E-04** | 8.76E-06 – 4.90E-02  8.33E-03 ± 1.59E-02  **1.22E-03** | 1.10E-04 – 3.77E-02  1.04E-02 ± 1.41E-02  **2.83E-03** | H= 1.776 | 0.620 |
| **P/B Ratio**  Min. – Max.  Mean ± SD.  Median | 0.00 – 88.58  14.25 ± 28.96  **0.62** | 0.00 – 0.10  0.05 ± 0.05  **0.03** | 0.00 – 14.23  3.56 ± 5.67  **0.10** | 0.00 – 19.00  2.92 ± 6.66  **0.01** | H= 2.911 | 0.406 |
| **F/B ratio** |  |  |  |  |  | **0.015^*^** |
| Min. – Max. | 0.00 – 0.66 | 0.69 – 3.61 | 0.10 – 1.91 | 0.28 – 5.65 | H= 10.404^*^ |  |
| Mean ± SD. | 0.40 ± 0.20 | 1.94 ± 1.51 | 0.80 ± 0.62 | 1.74 ± 1.70 |  |  |
| Median | **0.40** | **1.51** | **0.67** | **1.32** |  |  |
| **Diversity index** |  |  |  |  |  |  |
| Min. – Max. | 1.01 – 1.46 | 1.11 – 1.31 | 0.91 – 1.43 | 0.97 – 1.43 | F=  0.170 | 0.915 |
| Mean ± SD. | 1.20 ± 0.12 | 1.21 ± 0.10 | 1.22 ± 0.19 | 1.26 ± 0.15 |  |  |
| Median | **1.21** | **1.22** | **1.31** | **1.27** |  |  |
| **Dissimilarity index (%)** |  |  |  |  |  |  |
| Min. – Max. | 24.00 – 66.00 | 23.00 – 31.00 | 22.00 – 92.00 | 21.00 – 46.00 | H= 7.689 | 0.053 |
| Mean ± SD. | 43.44 ± 11.61 | 26.67 ± 4.04 | 42.30 ± 20.46 | 32.00 ± 8.47 |  |  |
| Median | **43.00** | **26.00** | **36.00** | **31.00** |  |  |

F: F for ANOVA test H: H for Kruskal Wallis test

p: p value for association between different categories *: Statistically significant at p ≤ 0.05

**Table ( S 6 ):** Correlation between Disease duration and Gut microbiome in PD group

| **Gut microbiome** | **Disease duration** | |
| --- | --- | --- |
|  | **r** | **p** |
| ***Bacteroides*** | 0.119 | 0.530 |
| ***Prevotella*** | -0.344 | 0.063 |
| ***Ruminococcus*** | **0.387*** | **0.035*** |
| ***Firmicutes*** | -0.092 | 0.628 |
| ***Bacteroidetes*** | -0.010 | 0.960 |
| ***Lactobacilli*** | -0.262 | 0.162 |
| ***Bifidobacteria*** | 0.076 | 0.689 |
| **P/B Ratio** | -0.256 | 0.172 |
| **F/B ratio** | -0.094 | 0.622 |

**r: Pearson coefficient**
